# Characterization and evaluation of the ability of graphene quantum dots to affect α-synuclein aggregation in synucleinopathy models

**DOI:** 10.1080/14686996.2026.2662693

**Published:** 2026-05-07

**Authors:** Tuba Oz, Anna Alwani, Agnieszka Kamińska, Barbara Jachimska, Makoto Timmon Tanaka, Yasuo Miki, Koichi Wakabayashi, Katarzyna Maziarz, Sheetal Kaushik Bhardwaj, Ajeet Kaushik, Małgorzata Figiel, Piotr Chmielarz, Małgorzata Kujawska

**Affiliations:** a Department of Toxicology, Poznan University of Medical Sciences, Poznań, Poland; b Doctoral School, Poznan University of Medical Sciences, Poznan, Poland; c Department of Brain Biochemistry, Maj Institute of Pharmacology, Polish Academy of Sciences, Kraków, Poland; d Jerzy Haber Institute of Catalysis and Surface Chemistry, Polish Academy of Sciences, Kraków, Poland; e Department of Neuropathology, Hirosaki University Graduate School of Medicine, Hirosaki, Japan; f Van’t Hoff Institute for Molecular Sciences, University of Amsterdam, Amsterdam, The Netherlands; g Department of Environmental Engineering, Florida Polytechnic University, Lakeland, FL, USA; h Department of Biochemistry, Biophysics and Biotechnology, Jagiellonian University, Kraków, Poland

**Keywords:** Graphene quantum dots, bioactive nanomaterials, Nano–bio interactions, amyloid inhibition, nanotoxicology, cytocompatibility, nano–bio interface

## Abstract

Synucleinopathies, including Parkinson’s disease and multiple system atrophy (MSA), are neurodegenerative disorders characterized by aggregation of α-synuclein (ASN). Nanomaterials capable of modulating protein misfolding represent a potential intervention strategy. Here, we synthesized graphene quantum dots (GQDs) and systematically evaluated their physicochemical properties and biological activity against ASN aggregation. The GQDs were characterized using spectroscopic, electron microscopy, and colloidal techniques to determine surface chemistry, charge, optical properties, and crystalline structure. Biological evaluation demonstrated cytocompatibility in human dermal fibroblasts (IC_5__0_ = 90 µg mL^−1^ at 24 h) with assessments of DNA damage and inflammatory responses. Functionally, GQDs destabilized preformed ASN fibrils in a cell-free assay, as evidenced by reduced Thioflavin-T fluorescence. In primary murine dopaminergic neurons, GQDs decrease pS129-ASN inclusion formation without compromising neuronal viability. Most importantly, intranasal administration of GQDs in an MSA mouse model reduced ASN immunoreactivity in the brain. Collectively, our data indicate that the synthetized GQDs are bioactive and can modulate ASN aggregation across cell-free, neuronal, and in vivo models. Importantly, physicochemical properties govern nano – bio interactions, providing a rationale for further refinement of GQDs as a biomaterial platform for synucleinopathy-related applications.

## IMPACT STATEMENT

Graphene quantum dots developed in this study destabilize α-synuclein fibrils in vitro and attenuate α-synuclein pathology in a mouse model of multiple system atrophy, suggesting a promising direction for synucleinopathy research.

## GRAPHICAL ABSTRACT

**Figure:**
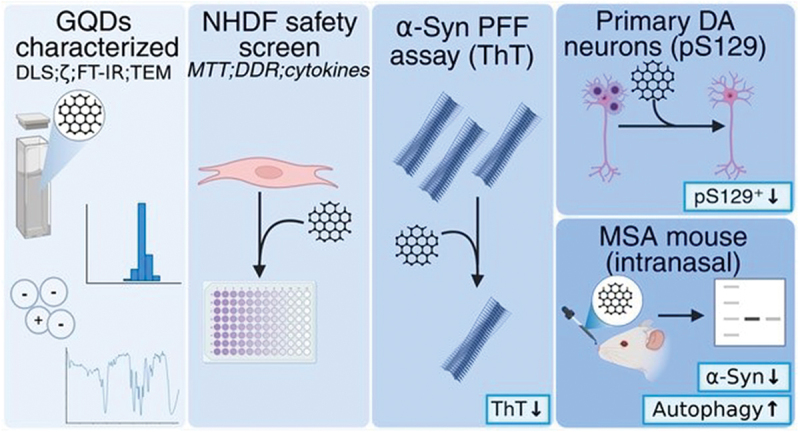

## Introduction

Among neurodegenerative diseases, synucleinopathies appear to be attractive targets for nanomaterial-based interventions due to their hallmark pathology – the misfolding and aggregation of α-synuclein (ASN), which can be modulated by bioactive nanomaterials. In this context, Graphene Quantum Dots (GQDs) are being explored as a bioactive nanomaterial capable of interfering with ASN assemblies and thereby affecting synucleinopathy-related pathology [1].

ASN is a relatively small, ~14 kDa presynaptic neuronal protein [2] that, despite being intrinsically disordered, can adopt α-helical conformations upon membrane binding [3]. While its physiological role includes modulating synaptic vesicle fusion and neurotransmitter release [4], its contribution to pathology lies in its propensity to misfold into flat, beta-sheet-rich amyloid protofilaments, which subsequently stack into twisted, elongated fibrils [5]. These aberrant aggregates are the primary component of intracellular inclusions characteristic of synucleinopathies – a major class of progressive neurodegenerative disorders that includes Parkinson’s disease (PD), dementia with Lewy bodies (DLB), and multiple system atrophy (MSA). In PD and DLB, ASN aggregates form Lewy bodies (LBs) and Lewy neurites in neurons. In MSA, in contrast, the predominant pathology involves glial cytoplasmic inclusions, also known as Papp-Lantos inclusions, in oligodendrocytes [6,7].

Formation of LBs, the major histopathological hallmark of PD, is driven by misfolded, pathological forms of ASN [8]. Increasing evidence suggests that these aggregates can propagate in a prion-like manner through interconnected brain regions, contributing to the disease progression [9,10]. The anatomical distribution of LB pathology was initially described by Braak [10] and later refined into brain-first and body-first PD subtypes [11]. As PD advances, ASN aggregates cause multiple cellular dysfunctions and contribute to the gradual death of specific neuronal populations, most importantly dopamine neurons, over many years [12]. PD diagnosis typically occurs late, as motor symptoms only manifest after substantial loss of dopaminergic neurons in the substantia nigra, possibly decades after initial ASN misfolding [12,13]. Current research thus prioritizes targeting pathological ASN aggregation at early, pre-symptomatic stages with particular emphasis on safety and long-term tolerability [13]. MSA, with a combination of autonomic failure, Parkinsonism, and/or cerebellar ataxia, poses an even greater therapeutic challenge due to its rarity, heterogeneity, and aggressive clinical course [14]. Unlike PD, the origin of ASN in oligodendrocytes remains unclear, implicating a possible neuron-to-glia transfer of pathological species [15]. MSA lacks effective disease-modifying therapies, and its rapid course reinforces the need for interventions that can interfere with ASN aggregation and spread at early stages [16].

Remarkably, GQDs have recently been demonstrated as promising agents capable of preventing ASN aggregation in test tubes, cultured cells, and live animals [17]. Graphene, a two-dimensional carbon nanomaterial, has attracted widespread interest in materials science due to its extraordinary electrical, optical, and mechanical properties [18]. GQDs, nanoscale (<20 nm) zero-dimensional derivatives of graphene, are typically functionalized with oxygen- and hydrogen-containing groups (e.g. –COOH,–COC–,–OH), which confer aqueous solubility, surface modifiability, and biointeractivity [19–21]. Their unique physicochemical profile: strong photoluminescence, high surface area with tunable chemistry, and remarkable biocompatibility positions GQDs as promising candidates for biomedical applications [19–21]. In vitro and in vivo studies have shown that GQDs possess minimal toxicity, are non-accumulative in major organs, and are rapidly excreted via renal pathways [22]. Shang *et al*. demonstrated the absence of cytotoxic effects on neural stem cells, supporting their applicability in neural environments [23]. However, variability in GQDs physicochemical size, surface charge, and functionalization can hinder their reproducibility, clarity on how they act, and translational scalability. Nonetheless, considering their neural compatibility, modifiability, and minimal toxicity, GQDs have gained attention as an engineerable nanomaterial platform being investigated in neurodegenerative proteinopathies [1].

While in vivo data are still limited, experimental and computational studies have explored the therapeutic efficacy of GQDs [1,24]. Engineered GQD nanocomposites have been shown to cross the blood-brain barrier and disaggregate ASN fibrils (PFFs), preventing neuronal death in murine models [17,25]. Beyond aggregation inhibition, GQDs modulate oxidative stress, suppress neuroinflammation via immune modulation, and mitigate neuronal excitotoxicity features critically relevant to the multifactorial pathophysiology of synucleinopathies [26–28].

While these properties are promising, the underlying therapeutic mechanisms remain insufficiently understood. Inconsistencies in synthesis protocols and physicochemical characterization of GQDs complicate reproducibility and hinder cross-study comparisons. Moreover, most available data are derived from computational models and biochemical studies with limited validation in disease-relevant contexts. This significant gap in translational evidence highlights the need for systematic, multidisciplinary research to clarify the therapeutic potential of GQDs in disease-relevant models.

Here, we examined the biocompatibility, anti-aggregative effects, and neuroprotective potential of custom-synthesized GQDs in a set of complementary experimental models: human dermal fibroblasts (NHDF) cells, biochemical aggregation assays, primary dopaminergic neuron models, and an in vivo mouse model of MSA, respectively. We hypothesize that GQDs synthesized for this study can mitigate ASN pathology via direct interaction with fibrillar species and modulation of autophagy. By integrating physicochemical characterization, cytotoxicity profiling, and functional assessment in vitro and in vivo, this study seeks to establish a basis for understanding the multimodal activity of GQDs in the context of synucleinopathy. This work provides a basis for further refinement of nanomaterial-based strategies targeting protein aggregation and related neurodegenerative mechanisms.

## Methods

### Synthesis of GQDs

Multilayer graphene oxide (GO) sheets were ultrasonically exfoliated into monolayer GO sheets in an ultrasonic cleaner for 12–14 h. Monolayer GO sheets were then thermally reduced into expanded graphene sheets (GSs) at 600°C (a heating rate of 10°C min) for 4 h in a nitrogen atmosphere. GSs (5 mg) were oxidized in concentrated H_2_SO_4_ (10 mL) and HNO_3_ (30 mL) for 18–20 h under ultrasonic conditions using the ultrasonic cleaner. After the oxidation, the mixture was diluted with deionized water (20 mL), divided into two centrifuge tubes (50 mL), and centrifuged more than 3 times to remove the acids. The oxidized GSs were dissolved in deionized water (40 mL) with pH tuned to > 12 with 4 mL of NH_3_·H_2_O and hydrothermally treated in a polytetrafluoroethylene Teflon-lined autoclave (50 mL) at 200°C for 10–12 h. After being cooled down to room temperature, the product was filtered through a 0.22 μm microporous membrane. A light-yellow supernatant containing fluorescent GQDs was obtained after the filtered solution was concentrated and centrifuged.

### Characterization of synthesized GQDs

#### Measurements of size and electrophoretic mobility

The size (hydrodynamic diameter) of GQDs was determined using the dynamic light scattering (DLS) method with the Malvern Zetasizer Nano ZS instrument, which is equipped with a 4 mW He – Ne laser operating at a wavelength of 633 nm and a fixed detector angle of 173°. The average hydrodynamic diameter and the polydispersity index were determined from the diffusion coefficient of particles subjected to Brownian motion. Measurements were performed 10 times.

The GQDs sample was diluted in water to a final concentration of 10 ppm and, directly before the measurement, was sonicated for 2 minutes (cycle: 0.5, amplitude: 60%). The hydrodynamic diameter of GQDs was calculated using the Stokes-Einstein equation:

$$R_{H}=\frac{\mathrm{kT}}{6\mathrm{\pi \eta D}}$$

where: k-Boltzmann constant; T-absolute temperature; η-viscosity of medium; and D-diffusion coefficient.

The same instrument was applied to measure the zeta potential of GQDs using laser Doppler velocimetry (LDV) technique. Measurements were performed 5 times. The zeta potential was measured for GQDs in concentration 100 ppm and was calculated using Henry’s equation:ζ

$$\xi =\frac{3\eta \mu_{e}}{2\mathrm{\varepsilon f}\left(\mathrm{\kappa a}\right)}$$

where: ζ is zeta potential, ε is the dielectric constant of water, η is the solution viscosity, f(κa) is the function of the dimensionless parameter кa.

#### Ultraviolet-visible (UV-Vis) spectroscopy

Ultraviolet-visible (UV-vis) spectra for the complexes were obtained using a Thermo Scientific Evolution 201 UV-Vis spectrophotometer in the wavelength range of 190–500 nm with a 2 nm slit width and a 1 cm path length, at intervals of 1 nm, with the solvent used as a baseline. UV-vis spectroscopy was used to measure the absorbance spectra of GQDs in a concentration range of 1–10 ppm.

#### Transmission electron microscopy (TEM)

GQDs were examined by transmission electron microscopy (TEM) to assess their morphology and crystalline structure. A drop of the GQD suspension was placed on a copper TEM grid coated with an ultrathin carbon film on a holey support and allowed to dry. Observations were performed using a JEM-ARM200F NEOARM microscope (JEOL, Japan) operated at 200 kV in bright field (BF) mode. Selected-area electron diffraction (SAED) patterns were obtained using a diffraction aperture and analyzed with Java Electron Microscopy Software (JEMS, developed by Pierre Stadelmann, Switzerland). High-resolution transmission electron microscopy (HRTEM) imaging was used to analyze the crystalline domains in detail. Images were recorded using JEOL CMOS and Gatan METRO cameras, and HRTEM image analysis was conducted using Digital Micrograph software (Gatan, U.S.A.).

#### Measurements of density and viscosity of GQDs

The density, dynamic, and kinematic viscosity of GQDs in concentration 10 ppm was measured using a capillary viscosimeter (DMA 5000 M, Anton Paar). The temperature of measurement was thermostated at 25°C.

#### Fourier transfer infrared (FT-IR) spectroscopy

Fourier transfer infrared (FT-IR) measurements were performed using a Nicolet iS50, Thermo Fisher Scientific, MA/U.S.A. FTIR spectrometer with a (SR) SMART SAGA attachment. FT-IR spectra were recorded in the wavenumber range from 400 to 4000 cm^−1^. Sample spectra were obtained by averaging 512 scans with a spectral resolution of 4 cm^−1^. Before each measurement, the spectrum of the initial surface was recorded and automatically subtracted from the sample spectrum. Omnic software (Thermo Fisher Scientific, MA/U.S.A.) was used to analyze the spectra. 20 μl of GQDs sample was mounted on the gold surface and left to dry.

#### Contact angle

The contact angle measurements were performed using an axisymmetric drop shape analysis (ADSA) system with an accuracy of 1°. For the contact angle measurements, GQDs in concentrations 10, 100 and 1000 ppm were placed on the gold surface and left for dry. The image of a drop of sitting water was obtained with a CCD camera, and the drop’s shape on the sensor surface was fitted by the Young-Laplace equation. The contact angle values were calculated as the average value of 5 measurements for each GQDs concentration, and the measurement error was defined as the standard deviation from the average.

### Biocompatibility studies in vitro

The biocompatibility studies in vitro were conducted using a validated experimental framework described previously [29], ensuring reproducibility and methodological consistency.

#### Cell culture

NHDF cells (PromoCell, C-23210, Heidelberg, Germany) were cultured in Dulbecco’s Modified Eagle Medium (DMEM, Sigma, D0819) supplemented with 10% fetal bovine serum (FBS; Sigma, Lot: 0001653683), 1 µg mL^−1^ penicillin-streptomycin (Sigma, Lot: 0000191002), and 2 mM L-glutamine (Sigma, RNBL6712). Cultures were maintained at 37°C in a humidified 5% CO_2_ atmosphere and sub-cultured upon reaching 80–90% confluency.

#### Measurement of in vitro cellular cytotoxicity

The cytotoxicity of GQDs was assessed via 3-[4,5-dimethylthiazol-2-yl]-2,5 diphenyl tetrazolium bromide (MTT) assay (Cell Proliferation Kit I, Roche Diagnostics; cat. no. 11,465,007,001), performed in accordance with the manufacturer’s instructions. NHDF cells were seeded in 96-well plates at a density of 5 × 10^3^ cells/well and incubated for 24 h. Cells were then exposed to GQDs at concentrations ranging from 10 to 200 µg mL^−1^ for 24, 48, and 72 h. Following exposure, 10 μL of MTT reagent (0.5 µg mL^−1^ final concentration) was added to each well and incubated for 4 h. Subsequently, 100 μL of solubilization buffer (10% SDS in 0.01 M HCl) was added, and plates were incubated overnight. Absorbance was measured at 570 nm using an ELISA reader (BioTek, U.S.A.).

#### DNA damage response (DDR) profiling

The expression of 27 DNA damage-related proteins was evaluated using the RayBio® C-Series Human DNA Damage Response (DDR) Antibody Array 1 (RayBiotech Life, U.S.A.), according to the supplier’s protocol. NHDF cells (1 × 10^6^ cells/well) were seeded in 6-well plates and exposed to a cytotoxic dose of GQDs for 24 h. Post-treatment, cells were lysed using ice-cold lysis buffer supplemented with a protease inhibitor cocktail. Lysates were centrifuged at 14,000 × g for 5 min at 4°C, and protein concentrations were determined via the BCA assay (Sigma; BCA1, B9643). A total of 200 μg protein was applied per array. Detection involved sequential incubation with biotinylated antibodies, HRP-streptavidin, and chemiluminescent substrate. Signal acquisition was performed with the iBright Imaging System (Invitrogen), and densitometric analysis was conducted in ImageJ (NIH), normalized against internal positive controls.

#### Cytokine inflammation profiling

Secreted cytokines were analyzed using the Human Inflammation Array 3 Kit (RayBiotech, QAH-INF-3), which quantifies 40 inflammation-related proteins. NHDF cells were cultured in 6-well plates (1 × 10^6^ cells/well) and treated as described. After 24 h, supernatants were collected, diluted 1:2 in sample diluent, and applied to pre-blocked arrays. Arrays were incubated overnight at 4°C, followed by labeling with Cy3-streptavidin. Fluorescence signals were scanned with a laser scanner and analyzed using GenePix software with the manufacturer-provided GAL files (www.RayBiotech.com/Gal-Files.html).

### In vitro studies with preformed PFFs

#### PFFs production and characterization

PFFs were prepared utilizing the protocol described by Volpicelli-Daley et al. (2014) with modifications [30]. Recombinant mouse ASN was expressed in BL21 RIL cells transformed with a pD454-SR mouse ASN plasmid (Addgene, #89075). Protein expression was induced with 1 mM IPTG. Cells were disrupted by sonication and the bacterial lysate was precleared by boiling, and ASN was enriched by ion-exchange chromatography using Fractogel-TMAE (Merck) and MonoQ (Cytiva) columns, followed by purification by size-exclusion chromatography on a Superdex 75 column (GE Healthcare) equilibrated with 10 mM Tris-HCl (pH 7.4), 10 mM Na_2_HPO_4_, 100 mM NaCl. Obtained ASN monomer was further purified with Pierce High-Capacity Endotoxin Removal Spin Columns (ThermoScientific, 88,274). To obtain fibrils, ASN monomers were aggregated by incubation with shaking at 37°C for 7 days as described in established protocols [31]. For cell culture experiments in dopamine neurons, the resulting PFFs were diluted in 1×PBS to a final concentration of 100 µg mL^−1^ and sonicated using a probe sonicator (Hielscher UP100H, 2 mm probe, 2 min, 80% amplitude, 0.5 s cycle). The presence of amyloid structure and PFFs quality was verified by thioflavin T assay and Circular Dichroism measurements.

#### Cell-free Thioflavin T (ThT) fluorescence assay

Thioflavin T (ThT) fluorescence assay was employed to semi-quantitatively evaluate the extent of fibrillization of PFFs in the presence of the tested GQDs. The assay was conducted in black 384-well microplates (Greiner) by mixing a ThT working solution (25 µM in PBS) with PFFs, with or without GQDs, under defined experimental conditions. To distinguish potential interactions between GQDs and different conformational states of ASN, separate sets of experiments were performed: one involving PFFs with or without GQDs, and another involving monomeric ASN with or without GQDs. Additional controls included GQDs alone and buffer-only samples. Fluorescence intensity was measured at three time points: immediately after mixing, after 24 h, and after 7 days of incubation at room temperature (excitation/emission: 450/480 nm). The assay enabled detection of β-sheet-rich fibrillar structures based on characteristic increases in ThT fluorescence relative to appropriate controls.

### Studies in dopaminergic neurons

#### Mice primary dopaminergic cultures preparation

Primary dopaminergic neuronal cultures were prepared according to previously published protocols [32]. Embryonic midbrain area was dissected from Albino Swiss embryos at embryonic day 13–14 (E13/E14) and washed 3 times with cold 1x w/o Ca^2+^ and Mg^2+^. Next, the 17-minute incubation with 0,25% trypsin at 37°C was performed for tissue dissociation. Following incubation, the tissue was triturated with DNase I in HBSS w/o Ca^2+^, Mg^2+^ with 10% FBS for a single cell separation. Cell count and viability were automatically quantified using Cell Counter (Biorad), and cells were resuspended in DMEM/F12 medium (Thermo Scientific (Gibco) #21331–020, Waltham, MA, U.S.A.), supplemented with 5 µM L-Glutamine (Thermo Scientific (Gibco), #25030–032, Waltham, MA, U.S.A.), 1× N-2 serum supplement (Thermo Scientific, #17502–048, Waltham, MA, U.S.A.), 150 µM D-glucose (Sigma-Aldrich, #G8769, St. Louis, MO, U.S.A.) and 200 ng mL^−1^ Primocin (Invivogen; ant-pm-1, ant-pm-2, San Diego, CA, U.S.A.). Cells were plated in micro-islands at a density of 5 × 10^4^ cells/well of 96-well plates previously pre-coated with poly-L-ornithine (#P4538-500 MG).

#### Neuronal culture staining

Cells were first fixed with 4% paraformaldehyde for 15 min at room temperature, then washed 3 times with 1xPBS and permeabilized for 15 min with 0.2% Triton X-100. Next, a blocking solution containing 0.2% Triton X-100, 5% FBS in PBS was added and incubated for 1 h at room temperature. After removing the blocking solution, primary antibodies were added (Mouse anti-tyrosine hydroxylase (TH^+^), Millipore, #MAB318 1:2000; Rabbit anti-pS129-ASN Abcam, # ab51253, 1:2000) and incubated overnight at 4°C. The following day, cells were washed 3 times with 1xPBS, and proper secondary antibodies were added, followed by incubation for 1 h at room temperature. Washed 3 times with 1xPBS, cells were ready for imaging.

#### Fluorescent imaging of neuronal cultures

The imaging of stained mouse dopaminergic neuronal cultures was performed using a Leica Thunder Microscope (Leica Microsystems) at 10x magnification. Nine images were captured per well of the 96-well plate, utilizing autofocus settings to maintain consistent focus. Fluorescent signal acquisition was optimized for each channel to minimize photobleaching and ensure clear visualization of neuronal morphology and ASN aggregates. Collected images were then processed using the open-access CellProfiler [33] software for automated image analysis, followed by CellProfiler Analyst [34] for machine learning-based classification, as previously described [32].

### In vivo study in MSA model

#### Animals

All animal experiments were approved by the Animal Research Committee of Hirosaki University (Approval No. AE01-2025–036) and conducted in accordance with institutional guidelines for animal care and use. Mice were housed in temperature- and humidity-controlled conditions under a 12 h light/dark cycle (lights on at 7:00 AM), with food and water provided ad libitum.

#### MSA mouse model

The MSA mouse model was generated by crossing human α-Syn-flox transgenic mice with the proteolipid protein-Cre recombinase/oestrogen receptor transgenic mice [34]. In this model, human ASN was overexpressed in oligodendrocytes after intraperitoneal tamoxifen injection (100 mg/kg/day ×five consecutive days) [35].

#### GQDs treatment protocol

Following induction, a subset of MSA model mice received intranasal administration of GQDs at a dose of 10 μg per mouse, twice weekly, for four weeks. The dose for GQDs was established experimentally based on biodistribution studies following intranasal administration of GO [36], and the dosing regimen was further optimized through pilot studies evaluating the delivery route and dosing parameters. GQDs were suspended in sterile PBS and homogenized using a probe-type ultrasonic sonicator immediately prior to each administration to ensure uniform dispersion. Each experimental group consisted of five animals. In parallel, vehicle-treated MSA mice and age-matched wild-type controls received PBS via the same intranasal route.

#### Immunohistochemistry and western blotting for ASN

At the end of the 4-week treatment period, mice were euthanized, and their brains were dissected. One hemisphere was fixed in 4% paraformaldehyde overnight for immunohistochemical analysis (IHC), while the other was snap-frozen for Western blotting (WB). The control mice (49.2 weeks of age), the MSA model mice with GQDs (54 weeks of age), and the MSA model mice without GQDs (54 weeks of age) were matched for age and sex. For IHC, 4-μm-thick, paraformaldehyde-fixed tissue was paraffin-embedded, and sagittal sections of the cerebellum were dewaxed, rehydrated, and subjected to heat-induced antigen retrieval in citrate buffer (pH 6.0). Endogenous peroxidase activity and nonspecific binding were blocked prior to overnight incubation at 4°C with a monoclonal anti-ASN antibody (clone 211, Abcam). Detection was achieved using a biotinylated secondary antibody and DAB substrate (Sigma-Aldrich).

For Western blotting, snap-frozen brain tissue was homogenized and lysed in RIPA buffer supplemented with protease inhibitors. Protein concentrations were determined using the BCA assay (Sigma; BCA1, B9643). Equal amounts of total protein were separated on 8–12% SDS-PAGE gels and transferred onto polyvinylidene fluoride (PVDF) membranes. Following transfer, membranes were blocked in an appropriate blocking buffer and then incubated overnight at 4°C with the respective primary antibodies. The following primary antibodies were used: anti-α-synuclein (ab80627, Abcam; clone 211), anti-Beclin-1 (D40C5, Cell Signaling Technology), and anti-LC3 II/I (ab128025, Abcam) antibodies. For ASN detection, membranes were pre-treated with 4% paraformaldehyde in PBS overnight at 4°C before blocking. After incubation with the primary antibodies, membranes were washed and incubated with horseradish peroxidase (HRP)-conjugated secondary antibodies. Protein bands were visualized using enhanced chemiluminescence, and densitometric analysis was performed using ImageJ. All experiments were independently repeated three times.

### Statistical analysis

Statistical analyses were performed using GraphPad Prism 10.3.0 (GraphPad Software, Inc., La Jolla, CA, U.S.A.). Depending on data distribution and experimental design, appropriate statistical tests were applied, including the Kruskal – Walli’s test with Dunn’s multiple comparisons test, one-way ANOVA with Dunnett’s, Sidak’s, Tukey’s, or Fisher’s LSD post-hoc tests, and unpaired Student’s t-test. Data are presented as either median ± IQR or mean ± SD, as indicated in the figure legends. A *p*-value < 0.05 was considered statistically significant. KEGG pathway-level modulation was calculated as log_2_ fold change (GQDs/Control) based on mean cytokine values.

## Results and discussion

### Characterization of synthesized GQDs

**Figure 1.**
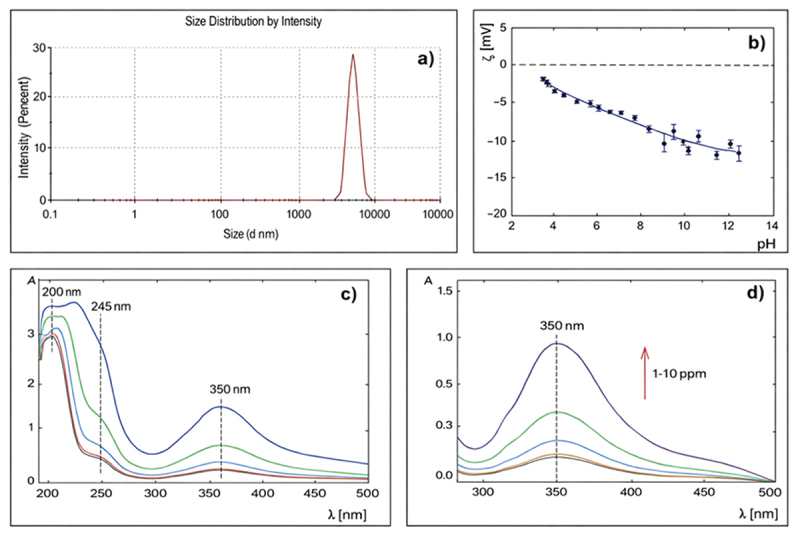
The size of GQDs. (A) Size distribution of GQDs in water, (B) zeta potential of GQDs in water solution, (C) UV-Vis spectra in the range from 200–500 nm for GQDs in water for a concentration 1–10 ppm, (D) UV-Vis spectra in the range from 280–500 nm for GQDs in water.

The size of GQDs was determined using the DLS method. As shown in Figure 1(a), a size distribution was obtained for GQDs dispersed in water at a concentration of 10 ppm at pH 3.95. The hydrodynamic diameter was 538 ± 77 nm, with a polydispersity index of 0.56. The DLS measurement shows that the GQDs tend to form aggregates.

The results of the zeta potential measurements of GQDs in water are summarized in Figure 1(b). The isoelectric point for GQDs occurs at a very low pH level <2. In the entire pH range, the molecules are negatively charged. For high pH, the zeta potential is about −12 mV. Since the charge of the molecules across the entire pH range is below −30 mV, the system tends to aggregate, as confirmed by DLS measurements. The effective charge of GQDs will have a key impact on the of the molecules in biological systems.

The optical properties of the GQDs were determined by recording the optical absorption spectra of the samples using a UV-vis spectrophotometer. Absorption spectra of GQDs diluted in water at a concentration of 1–10 ppm were presented in Figure 1(c–d). GQDs exhibited absorption at 200 nm, which is related to π electron transition from π to π* of C=C bonds in the aromatic domains of the graphitic structure [37], 350 nm which is attributed to the electron transitions from the π (or n) to π* orbital of C=O bond [38] and a typical π-π* transition absorption peak (due to the of aromatic sp 2 domains) around 250 nm [39].

**Figure 2.**
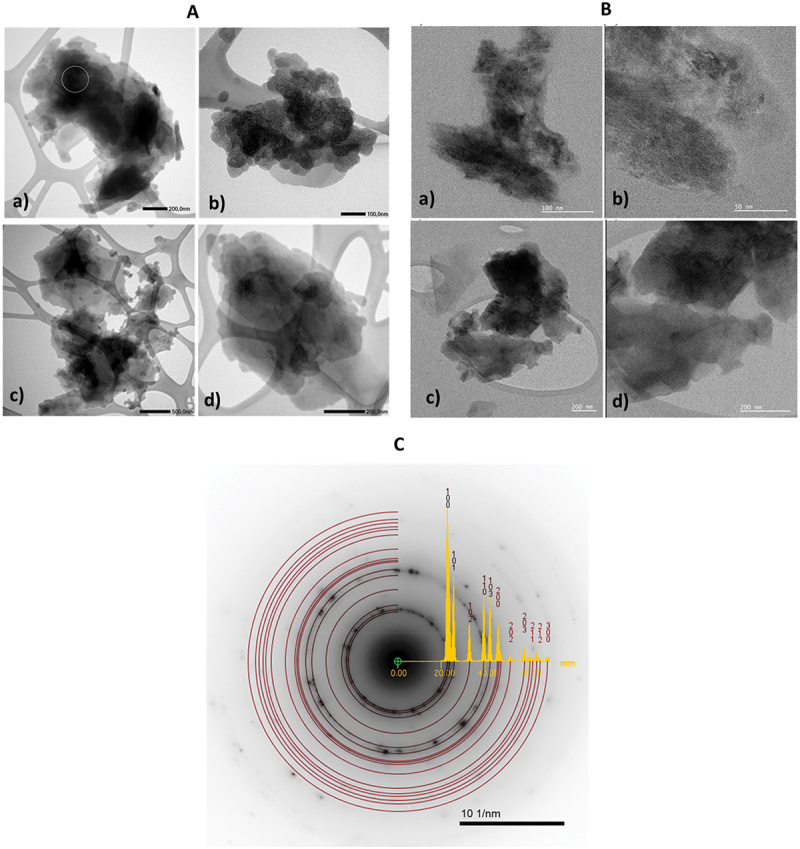
Bright field TEM image of GQD agglomerates recorded using a CMOS camera (JEOL). The circular region in panel (A) indicates the area from which the SAED pattern shown in Figure 2(B) was obtained. (B) Bright field TEM image of GQD flake aggregates recorded using the metro Gatan camera. The image illustrates the morphology and heterogeneity of the flake-like structures. (C) SAED pattern obtained from the area marked in Figure 2(A). Theoretical diffraction rings corresponding to hexagonal graphite (space group P6_3_/mmc) were superimposed on the experimental pattern using the JEMS software.

TEM revealed that GQDs formed agglomerates composed of stacked flake-like structures with a wide size distribution. The lateral dimensions of individual agglomerates ranged from approximately 130 nm up to 3000 nm. Within these aggregates, larger flakes (300–1000 nm) and smaller flakes (30–100 nm) were clearly distinguishable. Representative bright field TEM images recorded using JEOL and Gatan METRO cameras are presented in Figure 2(A,B), respectively, illustrating the morphology and heterogeneity of the flake aggregates.

SAED patterns were collected from the flake agglomerates, revealing concentric diffraction rings characteristic of a crystalline structure. A comparison of the measured ring positions with simulated diffraction patterns (Figure 2(C)) confirmed a good match with the hexagonal graphite phase (space group P6_3_/mmc, No. 194). However, the SAED patterns also exhibited weak individual diffraction spots and an additional ring between the (110) and (103) planes that could not be attributed to this phase. These features suggest the presence of structural disorder or minor unidentified crystalline components, which may be related to the functionalized or partially oxidized nature of the GQDs.

**Figure 3.**
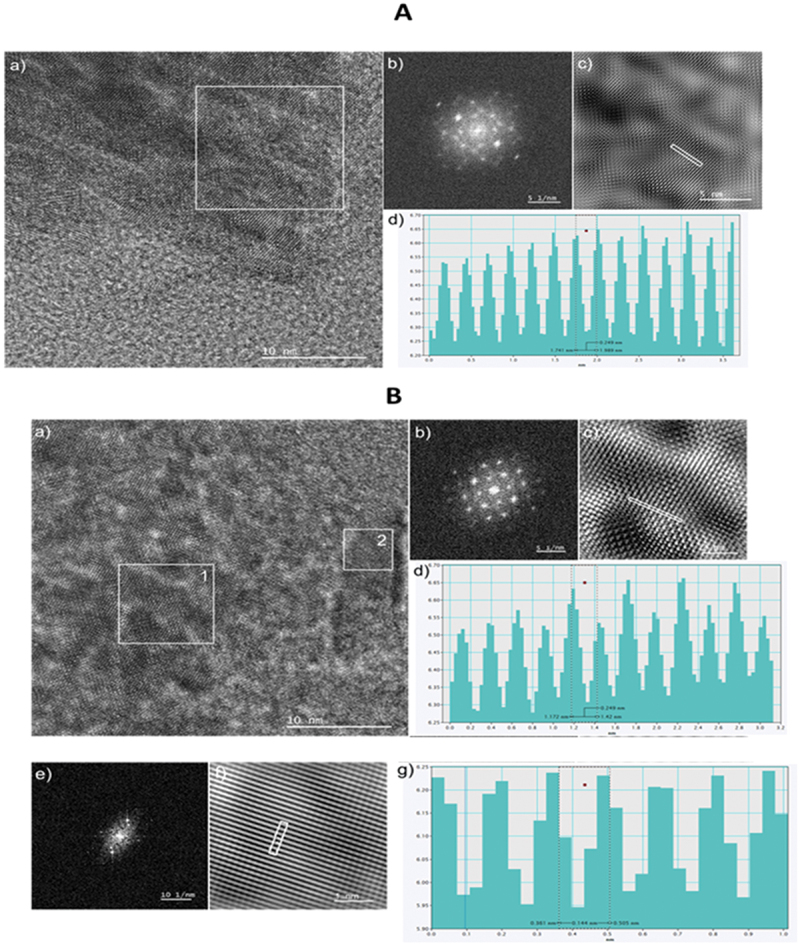
(A) HRTEM analysis of a GQD flake edge. (a) High-resolution TEM image; (b) FFT pattern from the region marked with a white square; (c) IFFT image obtained from the FFT with background filtering; (d) Interplanar spacing profile measured from the IFFT image. (B) HRTEM analysis of another region at the edge of a GQD flake. (a) High-resolution TEM image; (b, e) FFT patterns of the areas labeled 1 and 2, respectively; (c, f) corresponding IFFT images after background filtering; (d, g) Interplanar spacing profiles measured from the IFFT images in (c) and (f), respectively.

HRTEM was used to investigate the material’s crystallinity at the atomic level. HRTEM images (Figure 3(A,B)) taken at the edges of the flake agglomerates revealed individual graphene layers and localized lattice fringes. Fast Fourier Transform (FFT) and Inverse Fast Fourier Transform (IFFT) analyses were performed on selected regions to extract interplanar spacings dhkl, The measured d-spacings were 0.249 nm (Figure 3(A)) and 0.249 nm and 0.505 nm (Figure 3(B)). Interestingly, these values do not correspond to any of the characteristic planes of crystalline graphite previously identified by SAED. This discrepancy suggests that the GQDs contain locally ordered domains with lattice parameters deviating from ideal graphite, possibly due to the quantum confinement effect, the presence of defects, or oxygen-containing functional groups disrupting the long-range order.

In summary, the combined TEM, SAED, and HRTEM analyses confirm that the synthesized GQDs consist of crystalline graphene-like domains organized in flake-shaped aggregates. While SAED patterns point to a dominant hexagonal graphite-like structure, local HRTEM analysis reveals structural features inconsistent with pure graphite, indicating a more complex nanoscale structure. These findings are in agreement with previous reports on GQDs, which often exhibit a combination of crystalline cores and amorphous or functionalized edges.

**Figure 4.**
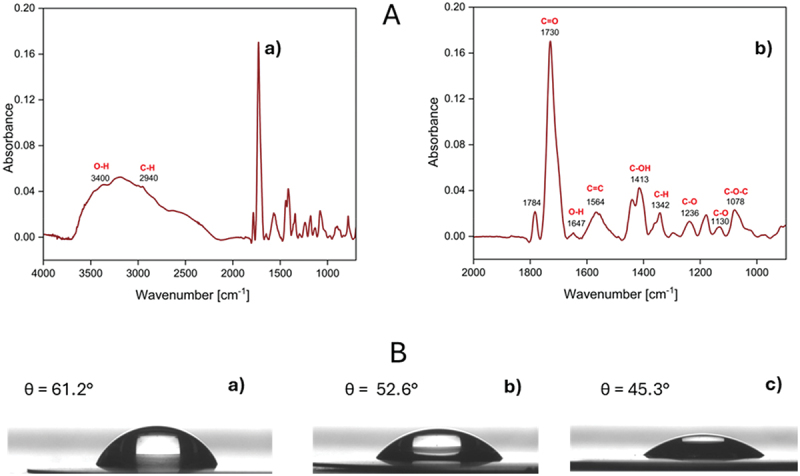
(A) FT-IR spectra of GQDs at a concentration of 50 ppm in the range (a) 4000–900 cm^−1^ and (b) 2000–900 cm^−1^. (B) Shape of a water drop placed on the GQDs layer on the gold surface for different concentrations of NP: (a) 10 ppm, (b) 100 ppm, (c) 1000 ppm.

FT-IR studies provided insights into the chemical composition, surface chemical bonds, and oxidation levels of nanoparticles. The characteristic FT-IR spectrum of graphene GQDs is depicted in Figure 4(A). The adsorption peaks can be assigned as follows: 1078 cm^−1^ to hydroxyl C-OH vibration, 1130 and 1236 cm^−1^ to C–O stretching vibrations, 1342 cm^−1^ to C-H groups, 1413 cm^−1^ to C-OH deformation, 1564 cm^−1^ to C=C in-plane vibrations, 1730 cm^−1^ to C=O stretching of carboxylic and/or carbonyl moiety functional groups and 2940 cm^−1^ to asymmetric stretching vibration of C-H bond [40–42], 3400 cm^−1^ to O-H.

The density of GQDs in water (c = 10ppm) was equal to 0,9969 ± 0,000027 g cm^3^, dynamic viscosity was equal to 0,8828 ± 0,000058 mPas, and kinematic viscosity was equal to 0,8856 ± 0.000058 mm^2/s^ (*T* = 25°C). The hydrophobic properties of GQDs molecules were determined for the layer adsorbed on the gold surface. The initial gold surface has a contact angle of 69.6 ± 0.4°. Adsorption of nanoparticles causes a decrease in the contact angle. For the lowest concentration, it is 61.2 ± 1.6°. With the increase in the concentration of nanoparticles, the contact angle decreases. For GQDs concentrations of 100 ppm and 1000 ppm, the contact angle was equal to 52.6 ± 2.3 and 45.3 ± 5, respectively (Figure 4(B)). Based on these measurements, it can be concluded that GQD particles have a hydrophilic character.

### Biocompatibility of GQDs

A comprehensive in vitro biocompatibility assessment of GQDs was conducted using NHDF cells. The evaluation included assays for general cytotoxicity (MTT assay), determination of IC_5__0_ values, DDR profiling, and analysis of the inflammatory response.

#### GQDs decreased cell viability in a dose-time-dependent manner in NHDF cells

**Figure 5.**
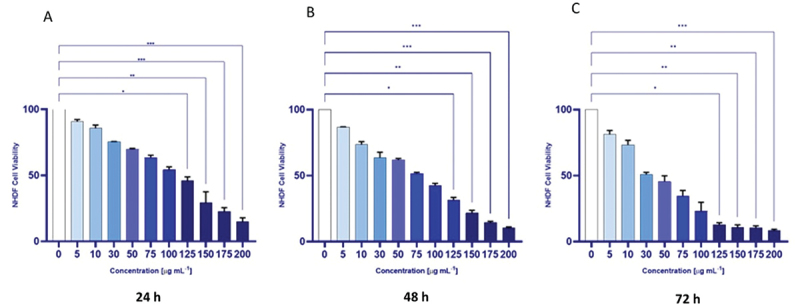
Viability of NHDF cells exposed to different concentrations of GQDs, as determined by the MTT assay, at 24 h (A), 48 h (B), and 72 h (C). Control cells were assigned 100% viability. The experiments were conducted in quadruplicate, and the data are expressed as median ± interquartile range (IQR). *p < 0.05, **p < 0.01, ***p < 0.001 by Kruskal – Wallis test with Dunn’s multiple comparison test.

As a first step, the cytotoxicity of GQDs towards NHDF cells was assessed using the MTT assay. Cells were treated with various concentrations of GQDs for 24 h, 48 h, and 72 h. Control cells were assigned 100% viability. Cells exposed to GQDs exhibited a concentration- and time-dependent decrease in viability (Figure 5).

Across all time points (24 h, 48 h, and 72 h), a significant reduction in cell viability was observed at GQDs concentrations of 125 µg mL^−1^ and above (*p* < 0.05). At lower concentrations (<125 µg mL^−1^), viability remained above 60% at 24 h, with a gradual decline over time. At 72 h, cells exposed to 125 µg mL^−1^ and higher concentrations displayed a pronounced cytotoxic effect, with viability dropping below 15% (*p* < 0.05). These findings align with previous studies demonstrating that GQD cytotoxicity is both dose- and time-dependent, with prolonged exposure typically exacerbating cellular damage. Liang et al. systematically reviewed experimental and computational studies and emphasized that cytotoxic effects of GQDs strongly correlated with exposure duration and concentration, as cumulative membrane disruption and oxidative stress intensify over time [43]. While GQDs are generally considered less cytotoxic than other graphene-based nanomaterials, their biocompatibility is not absolute and varies with physicochemical properties. For instance, Wu et al. demonstrated that GQDs exhibited significantly lower toxicity than micrometer-scale GO, exerting only mild effects on viability and cell cycle progression in MCF-7 and MGC-803 cells [44]. Similarly, Chong et al. reported minimal cytotoxicity of GQDs across several cell lines and in vivo models, even at relatively high concentrations [22]. Nevertheless, our results support prior evidence that the cytotoxic profile of GQDs is modulated by critical factors such as particle size, oxidative state, and surface functionalization [45]. In particular, elevated toxicity has been documented in lung carcinoma cells exposed to 100 µg mL^−1^ GQDs. Moreover, functionalization plays a pivotal role: hydroxylated GQDs (OH-GQDs) have been shown to induce significant cell death in A549 cells, whereas carboxylated GQDs (COOH-GQDs) demonstrated excellent biocompatibility [46]. These findings underscore the importance of careful physicochemical tuning of GQDs formulations to minimize potential adverse effects in biomedical applications. It should be noted that tested concentration range was very wide, and GQD doses at which toxic effects were observed are orders of magnitude higher than doses reported for GQDs to have a biological effect against ASN aggregation [17].

##### IC_5__0_ determination

**Figure 6.**
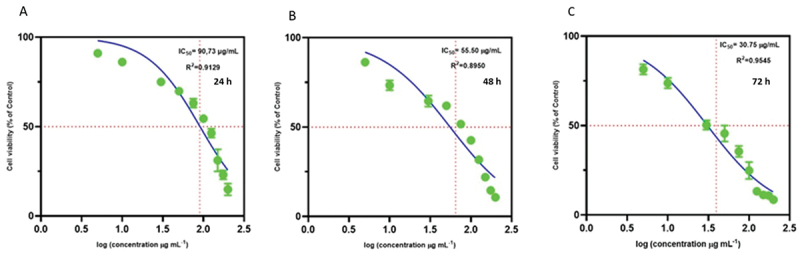
Ic_5__0_ values of GQDs in NHDF cells at 24 h (A), 48 h (B), and 72 h (C) are shown in panels A, B, and C, respectively. Data were analyzed using log-linear regression in GraphPad prism. Individual data points are shown in green (mean ± sd, where applicable). The blue curves represent the best-fit log-linear regression models. Each panel represents an independent dataset or experimental condition analyzed under the same fitting parameters.

The 50% inhibitory concentrations (IC_5__0_) of GQDs in NHDF cells were determined at 24 h, 48 h, and 72 h (Figure 6). The IC_5__0_ values were 90.73, 55.50, and 30.75 µg mL^−1^ at 24 h, 48 h, and 72 h, respectively. IC_5__0_ values decreased over time, indicating increased cytotoxicity with prolonged exposure. The calculated IC_5__0_ at 24 h was approximately 90 µg mL^−1^ and served as the reference concentration for subsequent mechanistic studies.

Reported IC_5__0_ values for GQDs span a broad range, reflecting their functional diversity. Pristine GQDs showed an IC_5__0_ of 24.81 µg mL^−1^ in MCF-7 cells [47], while functionalized variants exhibited selective toxicity: 78.8–89.7 µg mL^−1^ for cancer-targeted AGQD-NPs [48] and as low as 0.09 µg mL^−1^ in antiviral applications [49]. These findings highlight the need for careful optimization of GQD properties for safe biomedical use.

##### GQDs induced differential DDR pathway modulation in NHDF cells

**Figure 7.**
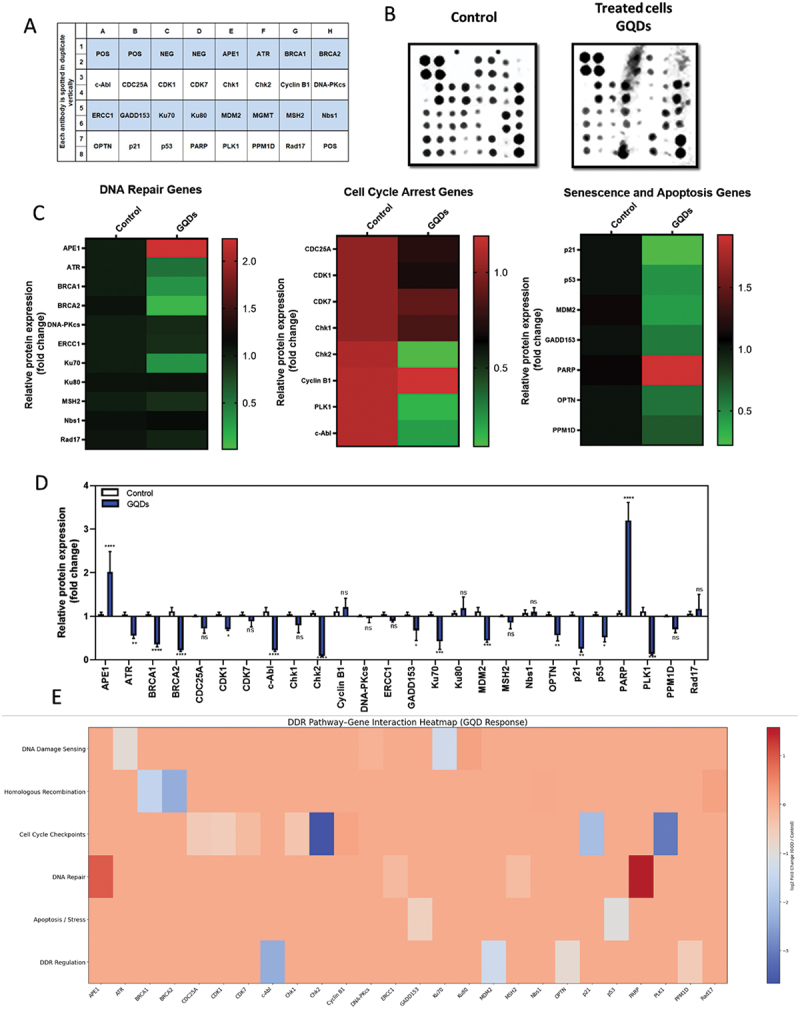
Expression profiles of DDR-related proteins in NHDF cells following 24 h exposure to GQDs at 90 µg mL^−1^. (A) Antibody array layout showing antigen-specific antibody spots; ‘nbs1’ control spots were used for data normalization, and ‘NEG’ spots served as negative controls for baseline signal measurement. (B) Representative images of the original antibody arrays. (C) heat map illustrating the relative expression levels of DDR-related proteins, with color intensity indicating normalized expression values. Data represent four independent experiments (*n* = 4). (D) semi-quantitative analysis of DDR-related proteins expression using antibody microarray in NHDF cells treated with GQDs at 90 µg mL^−1^ for 24 h. Data are expressed as mean ± standard deviation (sd) (*n* = 4) relative to control cells. Statistical significance was assessed using one-way ANOVA, followed by Sidak’s multiple comparisons test: ns: not significant, *p < 0.05, **p < 0.01, ***p < 0.001, ****p < 0.0001 vs. control. (e) GQDs induced differential DDR pathway modulation. Pathway–gene interaction heatmap visualized the relationship between key signaling pathways (rows) and DDR-related proteins (columns). Color scale denotes expression changes, with red indicating up-regulation, blue indicating down-regulation.

To investigate the potential genotoxic effects of GQDs, the expression profiles of DDR-related proteins were examined in NHDF cells following 24 h exposure to GQDs at IC_5__0_ concentration (90 µg mL^−1^). Representative images of the original antibody arrays and a heat map illustrating relative protein expression are presented in Figure 7. Quantitative analysis revealed that only two DDR-related proteins (APE1 and PARP) were significantly upregulated compared to control cells. Among the remaining proteins, several were significantly downregulated (ATR, BRCA1, BRCA2, CDK1, c-Abl, Chk2, GADD153, Ku70, MDM2, OPTN, p21, p53, PLK1), while others showed no statistically significant change (ns) (Figure 7(D)).

Overall, GQDs at IC_5__0_ concentration (90 µg mL^−1^) induced a selective modulation of DDR-related protein expression in NHDF cells. The significant upregulation of APE1 and PARP suggests activation of base excision repair and response to strand breaks, likely reflecting oxidative DNA damage – a mechanism previously observed for graphene-based nanomaterials, including both graphene oxide (GO) and GQDs [50,51]. In contrast, the downregulation of key proteins involved in checkpoint signaling (ATR, Chk2, p53, p21), homologous recombination (BRCA1/2), and non-homologous end joining (Ku70) indicates an altered regulation of DDR signaling networks [52,53].

Such a dual pattern – activation of selected DNA repair proteins alongside downregulation of multiple DDR regulators – reflects a complex cellular response to GQDs exposure. Pathway – gene interaction analysis further highlighted coordinated modulation of DNA repair, checkpoint control, and apoptosis-related signaling pathways following GQDs exposure (Figure 7(C,E)). Consistent with previous reports on ROS-mediated genotoxic effects of graphene derivatives [54,55] these results provide proteomic-level evidence of DDR pathway modulation and warrant further investigation into the long-term biological consequences of GQDs exposure.

##### GQDs induced inflammatory response

**Figure 8.**
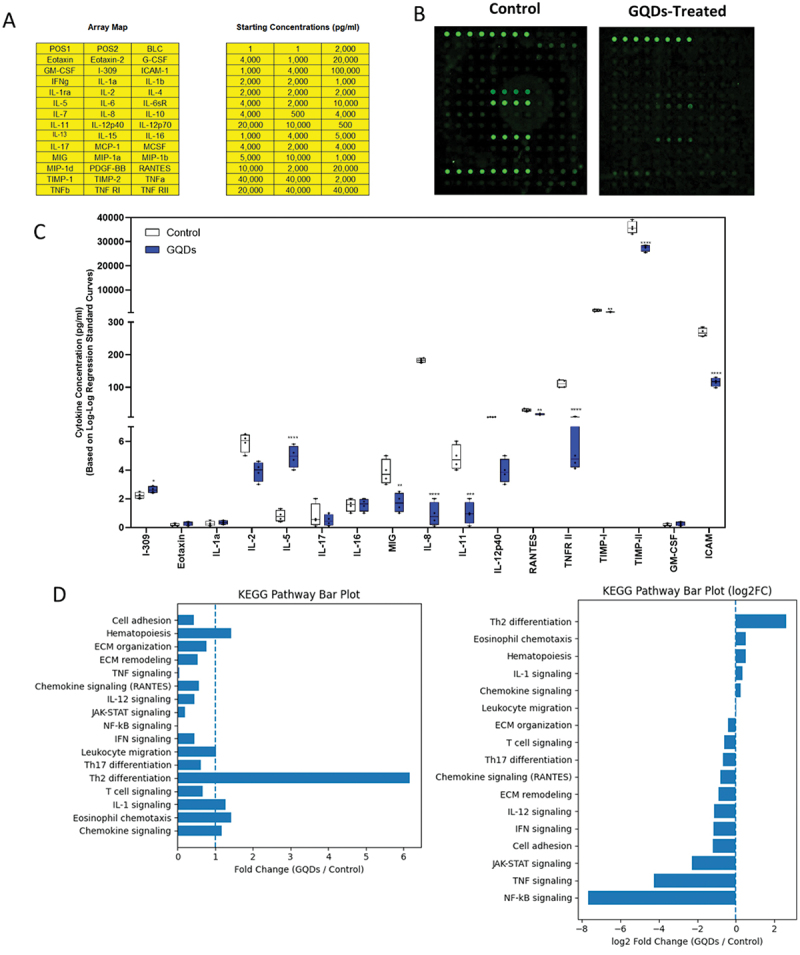
Expression profiles of cytokine-related factors in NHDF cells following 24 h treatment with GQDs at 90 µg mL^−1^. A human cytokine antibody array containing 40 cytokines was used, with ‘pos’ positive control spots applied for data normalization. (A) Each antibody was spotted in quadruplicate in a horizontal layout. (B) Representative fluorescence images of the cytokine antibody arrays. (C) Upregulated cytokines and downregulated cytokines were identified in NHDF cells treated with GQDs (90 µg mL^−1^, 24 h). The array detected two significantly upregulated cytokines compared to control cells. Eight cytokines were significantly downregulated, intercellular adhesion molecule, C-X-C motif chemokine 9, interleukins/, and metalloproteinase inhibitors. Statistical analysis was receptors performed using unpaired Student’s t-test. *p < 0.05, **p < 0.01, ***p < 0.005, ****p < 0.0001 vs. control. (D) Kyoto encyclopedia of genes and genomes (KEGG) showing immune pathway modulation following GQD exposure. Horizontal bar plot illustrating KEGG pathway enrichment based on log2 Fold change (GQDs/control). Negative values indicate pathway suppression and positive values indicate pathway activation following GQD exposure. GQDs markedly suppressed inflammatory and adhesion-related pathways while enhancing Th2-associated (IL-5) immune signaling.

The inflammatory response induced by GQDs was evaluated by profiling cytokine expression in NHDF cells treated for 24 h at IC_5__0_ concentration (90 µg mL^−1^). Representative fluorescence images of the cytokine antibody arrays are shown in Figure 8. Analysis revealed that two cytokines were significantly upregulated (I-309, IL-5) compared to control cells, while eight cytokines were significantly downregulated (MIG, IL-8, IL-11, RANTES, TNFRII, TIMP-I, TIMP-II, ICAM). Overall, exposure of NHDF cells to GQDs at IC_5__0_ concentration resulted in a selective modulation of inflammatory mediators (Figure 8(C)).

While I-309 (CCL1) and IL-5 were significantly upregulated, potentially reflecting a compensatory tissue-repair response [56], key pro-inflammatory cytokines (MIG/CXCL9, IL-8, IL-11, RANTES/CCL5) and matrix-regulating factors (TNFRII, TIMP-I/II, ICAM) were markedly downregulated. Pathway enrichment analysis further indicated reduced involvement of canonical inflammatory signaling pathways, including chemokine, cytokine – cytokine receptor interaction, and NF-κB – related pathways (Figure 8(D)). This pattern is consistent with previous reports that GQDs may elicit relatively low immunogenicity and ROS-mediated, but not strong pro-inflammatory, responses [55]. Given the role of chronic neuroinflammation in the progression of synucleinopathy, the observed modulation of inflammatory mediators warrants further investigation in disease-relevant experimental models.

##### GQDs led to a significant reduction in the stability of PFFs

**Figure 9.**
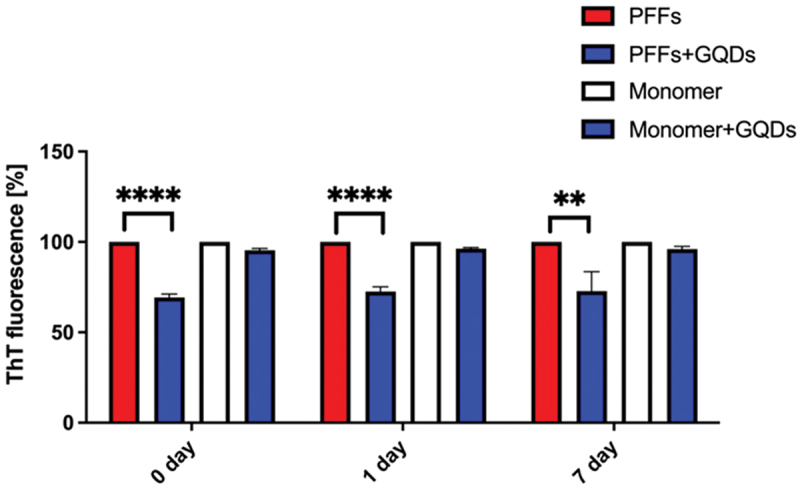
Kinetics of preformed PFFs and monomer after incubation with GQDs using aliquots of the reaction monitored by ThT fluorescence. Data are expressed as mean ± sd from five independent experiments. Fluorescence values were normalized to the respective control at each time point for PFF and monomer conditions, enabling comparison of temporal changes within each group. Note that this normalization does not reflect absolute differences in amyloid content between PFFs and monomer. *p < 0.01, **p < 0.0001 by unpaired t test.

The effect of GQDs on the stability of preformed PFFs was evaluated in vitro in microplate-based assays by monitoring ThT fluorescence over time. PFFs were incubated with GQDs in a microplate continuously for 7 days, and fluorescence measurements were performed on the same microplate at three time points (0, 1, and 7 days), without disturbing the samples. Data from five independent experiments are presented as mean ± SD (Figure 9).

Incubation of PFFs with GQDs resulted in a significant reduction in ThT fluorescence intensity compared to control samples (*p* < 0.001), indicating a decrease in fibril stability. Notably, the reduction in ThT fluorescence was consistent across all measured time points: fluorescence intensity was reduced by approximately 30% at 0 day, 1 day, and 7 days relative to untreated PFFs (*p* < 0.001, unpaired t-test). These results suggest that GQDs interact with PFFs and destabilize their structure, leading to impaired fibril stability under cell-free in vitro conditions. This finding aligns with previous reports highlighting the ability of graphene-based nanostructures to interfere with amyloid fibrillation and promote fibril destabilization through direct nanomaterial protein interactions [17,57]. The observed effect was already apparent at day 0 of incubation, suggesting that GQDs can rapidly engage with fibrillar ASN and alter its structural conformation. Importantly, the persistence of this destabilizing effect over 7 days indicates that GQD-PFFs interactions are not transient but result in a sustained impairment of fibril integrity. Mechanistically, such effects may arise from multiple interaction modalities, including π–π stacking, hydrophobic interactions, and hydrogen bonding between GQDs and exposed residues on the fibril surface [57]. These interactions may disrupt the β-sheet-rich architecture that underpins fibril stability, leading to partial disassembly of the fibrils. Since PFFs act as potent seeds driving pathological ASN propagation [58], their destabilization by GQDs may attenuate the seeding capacity and pathogenic potential of these species. Importantly, in our experimental conditions, incubation of recombinant mouse ASN monomer (purified under physiologically relevant conditions) with GQDs did not lead to an increase in ThT fluorescence, indicating that GQDs do not promote aggregation of physiologically functional ASN. This selective activity is highly desirable for therapeutic translation, as uncontrolled modulation of native ASN could pose safety concerns. In contrast, previous studies reported pro-aggregative effects of GQDs on the aggregation-prone A53T ASN mutant [59]. To explore whether such effects on fibril stability translate into functional neuroprotection, we next investigated the impact of GQDs on ASN aggregation and neuron survival in primary dopaminergic neurons.

##### GQDs reduced α-synuclein aggregation and neuron survival in primary dopaminergic neurons

**Figure 10.**
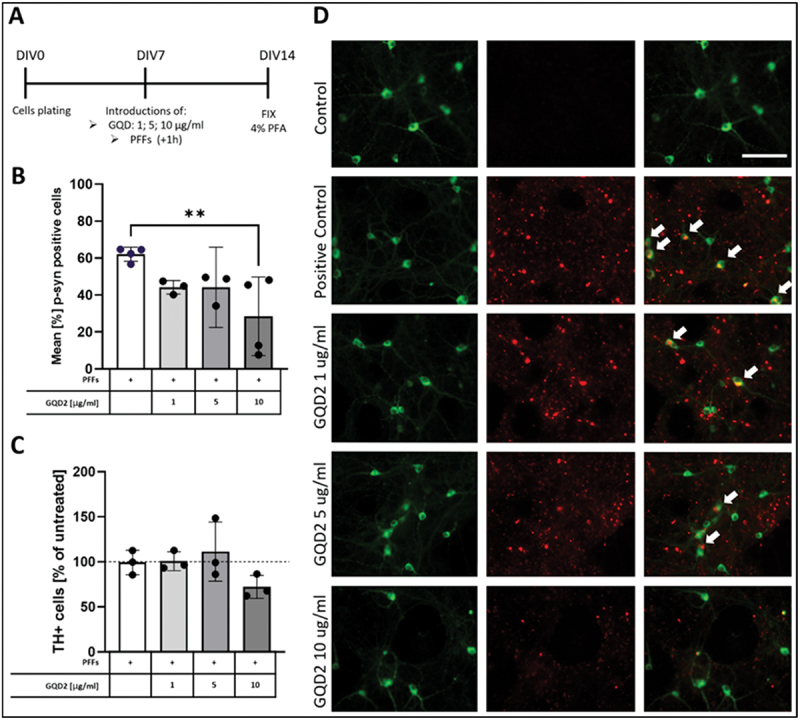
Gqds reduce ASN aggregation in primary dopaminergic neurons. (A) Experimental timeline: primary dopaminergic neurons were treated with GQDs (1, 5, or 10 µg mL^−1^) on DIV7, followed by PFFs 1 hour later. Cells were fixed and analyzed on DIV14. (B) Quantification of neurons with phosphorylated α-synuclein (pS129-α-syn) inclusions. GQDs treatment reduced the percentage of neurons with inclusions in a dose-dependent manner, with significant reduction at 10 µg mL^−1^ (**p < 0.01, Dunnett’s post-hoc test). (C) Quantification of TH^+^ neuron survival. No significant changes in neuronal survival were observed across treatments, although a slight decrease was noted at the highest GQDs concentration. (D) Representative immunofluorescence images showing th+ (green) and pS129-α-syn (red) staining. Scale bar = 50 μm. Data are presented as mean ± sd. *n* = 3 independent experiments.

To investigate the potential neuroprotective effects of GQDs against ASN pathology, we utilized an etiologically relevant primary dopaminergic neuron culture model exposed to preformed PFFs [32]. Neurons were treated with GQD at concentrations of 1, 5, and 10 µg mL^−1^, followed by PFFs introduction one hour later (Figure 10(A)). After 7 days, we assessed both the formation of phosphorylated ASN (pS129-ASN) inclusions in the cell soma of dopaminergic neurons and dopaminergic neuron survival.

PFFs treatment alone induced substantial pS129-ASN aggregation in the soma of TH^+^ dopaminergic neurons (Figure 10(B,C)). Approximately 62% of dopaminergic neurons contained substantial pS129-ASN inclusions in the positive control condition (Figure 10(B)). Notably, GQDs reduced the percentage of neurons with pS129-ASN inclusions in a dose-dependent manner, albeit only the highest dose at 10 µg mL^−1^, GQDs significantly decreased the proportion of affected neurons by more than half to about 28% (*p* = 0.0088, q = 4.331, DF = 7 for control vs. 10 µg mL^−1^ in Dunnets post-hoc test).

We also examined the effect of GQDs on dopaminergic neuron survival (Figure 10(D)) by quantifying TH^+^ cell number. As described by us and others previously [8,32], treatment with PFFs alone does not cause cell loss at the tested time point (7 days post-PFFs), which reflects the slow progressive nature of PD. Tested GQDs have not caused statistically significant loss of dopamine neurons; however, there was a tendency toward decreased survival in the highest GQDs concentration (72.19% vs 99.33% for 10 µg mL^−1^ vs PFFs-only group, *p* = 0.2299, q = 1.883, df = 7 in Dunnets post-hoc test).

Together, data from dopamine neurons show that the tested GQDs graphene formulation can effectively protect dopamine neurons from ASN aggregates, albeit the effect was significant at high doses – about 10× more than that reported for other formulations by Kim et al. [17] Survival data also suggest that such high concentrations might be to some extent detrimental to cells as vulnerable as dopamine neurons [60] consistently with our data from NHDF cells, therefore, further optimization of GQDs for higher efficacy and better biocompatibility should be considered for future therapeutic applications.

### In vivo efficacy in MSA model

The in vivo effects of GQDs intranasal treatment were evaluated in an MSA mouse model (synuclein-flox and Plp-Cre/ER) by analyzing ASN expression and autophagy markers. Normal mice served as controls.

#### GQDs significantly reduced ASN levels in the MSA mice

**Figure 11.**
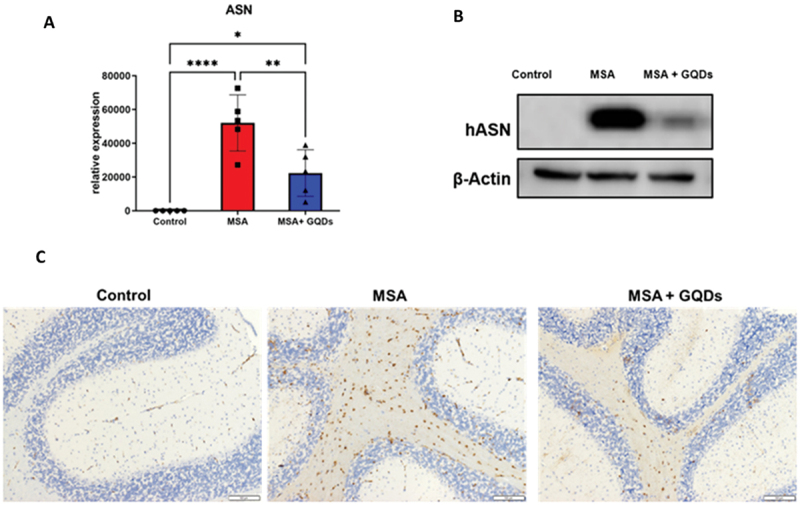
(A) Column graph and (B) representative Western blots present the quantitative Western blot analysis of human ASN expression in the cerebrum from normal mice and in mice with synuclein-flox and Plp-Cre/ER (MSA model). Data are expressed as the ratio of ASN to β-actin expression. Columns represent the mean ± sd of 5 animals per group. *p < 0.05, **p < 0.01, ***p < 0.001 by one-way ANOVA with a post hoc uncorrected Fisher’s LSD test. (C) representative images of human ASN immunostaining (syn211) in the cerebellum from normal mice and in mice with synuclein-flox and Plp-Cre/ER (MSA model).

The effect of GQDs treatment on ASN expression was assessed in the brain (cerebrum and cerebellum) of mice using the MSA mouse model (synuclein-flox and Plp-Cre/ER). Normal mice served as controls. Quantitative WB analysis demonstrated a significant increase in hASN expression in MSA mice compared to normal controls (*p* < 0.0001, Figure 11(A,B)). Treatment with GQDs significantly reduced ASN levels in the MSA mice by 40% (*p* < 0.01, Figure 11(A,B)).

These findings were corroborated by immunohistochemical analysis using syn211 antibody staining. Representative images showed pronounced ASN accumulation in the cerebellum of untreated MSA mice, which was visibly reduced following GQD treatment (Figure 11(C), right panel). Together, these results indicate that GQDs effectively reduce pathological ASN expression in the MSA mouse model.

In our study, GQDs significantly reduced cerebral hASN levels in MSA model mice (synuclein-flox × Plp-Cre/ER) by ~40%, counteracting the pathological overexpression characteristic of this oligodendroglial synucleinopathy. This finding provides in vivo evidence that GQDs modulate ASN burden in the brain, thus potentially attenuating a key molecular driver of disease progression in MSA. Our results are consistent with previous preclinical studies indicating that graphene-based nanostructures can interfere with pathological ASN processing in vivo. In the seminal work by Kim et al., GQDs were shown to cross the blood-brain barrier and disaggregate PFFs in multiple models of PD, including hA53T transgenic mice and PFFs-injected wild-type mice. Notably, systemic administration of GQDs reduced pS129-ASN immunoreactivity across several brain regions and attenuated behavioral deficits without inducing systemic toxicity [17]. Additionally, Kaliyaperumal et al. confirmed that functionalized GQDs not only inhibited ASN aggregation but also promoted defibrillation of preformed fibrils via interactions with ASN N-terminal residues. This defibrillation is associated with restored neuronal function and attenuated neurotoxicity in vitro and in vivo [25]. Our in vitro results align with these findings: we observed a dose-dependent reduction in the number of dopaminergic neurons harboring pathological pS129-ASN aggregates following GQDs treatment, without significant toxicity at effective concentrations. The convergence of in vitro and in vivo effects suggests that GQDs may target key conformational states or cellular pathways involved in ASN accumulation, possibly through direct interactions with fibrillar species or modulation of intracellular handling and trafficking. The reduction in total hASN most likely reflects GQD-mediated disaggregation of pathological ASN, followed by its accelerated clearance through the autophagy pathway, as evidenced by the subsequent up-regulation of autophagy markers.

The inhibitory activity of GQDs against ASN aggregation observed across all experimental models is consistent with their physicochemical properties. UV-vis and FT-IR characterization confirmed the presence of hydroxyl, carboxyl, and aromatic surface groups, which can mediate π–π stacking, hydrogen bonding, and electrostatic interactions with ASN. These surface functionalities remain operative regardless of the aggregation state of GQDs, as their anti-amyloid activity depends primarily on surface chemistry rather than monodispersity alone [24,61], Notably, molecular dynamics simulations have shown that larger GQD particles adsorbed a greater number of protein residues with higher binding strength, inducing more pronounced structural perturbations [62]. Systematic optimization of GQD dispersity and direct assessment of aggregated versus monodispersed forms remain important directions for future work.

#### GQDs promoted autophagy in the MSA mice

**Figure 12.**
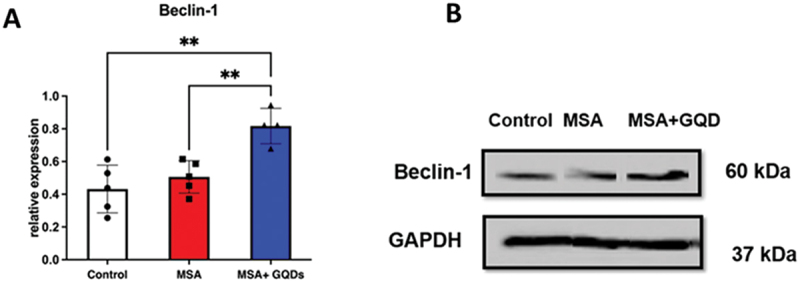
Column graph (A) and representative Western blots (B) present the quantitative Western blot analysis of Beclin-1 expression in the cerebrum from normal mice and in mice with synuclein-flox and Plp-Cre/ER (MSA model). Data are expressed as the ratio of Beclin-1 to GAPDH expression. Columns represent the mean ± sd of 5 animals per group. *p < .01 by one-way ANOVA with a post hoc Tukey’s multiple comparisons test.

**Figure 13.**
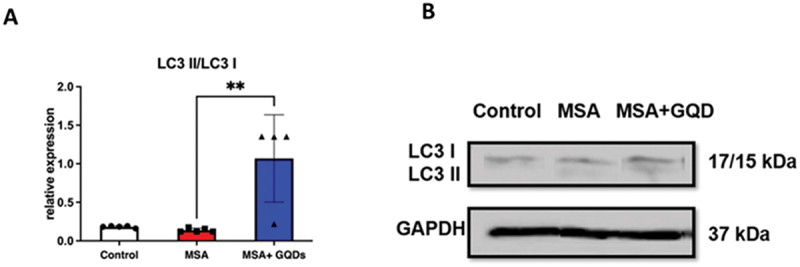
Column graph (A) and representative Western blots (B) present the ratio of the quantitative Western blot analysis of LC3 II/LC3 I expression in the cerebrum from normal mice and in mice with synuclein-flox and Plp-Cre/ER (MSA model). Columns represent the mean ± sd of 5 animals per group. *p < .01 by Kruskal-Wallis test with a post hoc Dunn’s multiple comparisons test.

The impact of GQDs treatment on autophagy markers in the cerebrum was evaluated by analyzing Beclin-1 expression and the LC3 II/LC3 I ratio in the MSA mouse model. Western blot analysis showed that Beclin-1 expression was not significantly different between untreated MSA mice and normal controls (ns). However, treatment with GQDs resulted in a significant increase in Beclin-1 expression in the MSA model by 40% compared to untreated MSA mice (*p* < 0.01) (Figure 12(A,B)). Similarly, the LC3 II/LC3 I ratio was significantly increased by 90% in the GQD-treated MSA mice (*p* < 0.01) (Figures 13(A,B)), indicating enhanced autophagic activity.

These results suggest that GQDs promote autophagy in the MSA mouse model, as evidenced by the upregulation of key autophagy-related markers in the cerebrum. Notably, untreated MSA mice did not exhibit significant alterations in Beclin-1 expression or LC3 II/I ratio compared to controls, suggesting that baseline autophagic signaling was not inherently dysregulated in this model. Therefore, the observed autophagy activation appears to be a specific effect of GQDs. Given the pivotal role of impaired autophagy in ASN accumulation and proteostasis disruption in synucleinopathies [63], this modulation may represent a critical mechanism by which GQDs exert disease-modifying effects. Previous studies have shown that nanomaterials, including QDs and graphene-based structures, can modulate autophagy through various mechanisms depending on surface properties and biological context. Stern and Johnson discussed that nanomaterial autophagy interactions may either promote degradation as an adaptive response or impair autophagic flux, depending on the context, with possible consequences for neurodegenerative disease susceptibility [63]. Specifically, Wang et al. demonstrated that engineered GQDs protect dopaminergic cells against ASN-induced toxicity via Beclin-1-dependent autophagy activation [64]. Similarly, Xie et al. reported that aminated GQDs strongly induced autophagic signaling without triggering cytotoxicity, suggesting their potential as modulators of cellular stress responses [46]. GO has been demonstrated to ameliorate Aβ accumulation through upregulation of the autophagic response in Alzheimer’s disease mice [65] and accelerate its endosomal delivery to lysosomes in mice with postoperative cognitive dysfunction [66] Supporting these findings, Shen et al. showed that GO induced Beclin-1 and increased the ratio of LC3-II/I in colorectal cancer cells, indicating autophagy activation via the ROS-dependent AMPK/mTOR/ULK1 pathway [67]. Alternatively, and consistent with the fibril disassembly we documented in the in vitro ThT assay, the Beclin-1 up-regulation and higher LC3-II/I ratio may simply represent a secondary, adaptive response: once GQDs loosen pathogenic ASN aggregates, the newly generated, more soluble species could trigger or become more accessible to baseline autophagic machinery, which then appears ‘activated’ in our read-outs.

## Conclusions

The GQDs synthesized and characterized in this study influenced key pathological processes relevant to synucleinopathies, including ASN aggregation observed in a cell-free assay, primary dopaminergic neurons, and MSA mice, as well as autophagy in the MSA model. Our results consistently indicate that the tested GQDs can affect ASN pathology at multiple levels of biological complexity. At the same time, dose- and time-dependent cytotoxicity and activation of DNA damage responses in NHDFs should be considered.

## Perspectives

GQDs are emerging as promising nanostructures capable of modulating key mechanisms implicated in synucleinopathies [17] with experimental evidence suggesting multifunctional synucleinopathy-related applications [1,68]. The toxicity profile of GQDs is highly dependent on their physicochemical properties, including size, surface chemistry, and functional group composition, and differs substantially across experimental models and exposure conditions [43]. Our biocompatibility data demonstrated that GQDs at therapeutically relevant concentrations did not significantly compromise cell viability. Nevertheless, comprehensive long-term toxicological evaluation remains an essential prerequisite for any future pre-clinical development. Since their biological activity is closely linked to physicochemical parameters and given the observed cytotoxicity and DNA damage responses in certain models, limited brain penetration, and concerns regarding long-term biodistribution, clinical translation of GQDs remains challenging [69,70]. Advancing the GQDs synthesized and tested herein toward synucleinopathy-relevant applications will require tailored surface functionalization, reproducible synthesis, and systematic evaluation of safety and efficacy in relevant disease models. Future development should also tackle brain-specific delivery strategies and long-term safety profiling to unlock their translational potential.

## Data Availability

The data that support the findings of this study are available from the corresponding author upon reasonable request.
